# Medicaid Expansion and Medication Use Among U.S. Children with ASD or ADHD: A Repeated Cross-Sectional Analysis Comparing Before and During the COVID-19 Periods

**DOI:** 10.3390/healthcare14050684

**Published:** 2026-03-09

**Authors:** Florida Uzoaru, Michael A. Preston, Travis Loux, Levi Ross

**Affiliations:** 1 College of Nursing and Health Sciences, Southeastern Louisiana University, 500 W University Ave, Hammond, LA 70402, USA; 2 Department of Pharmacy Practice, Purdue University, 610 Purdue Mall, West Lafayette, IN 47907, USA; mapreston@purdue.edu; 3 College of Public Health and Social Justice, Saint Louis University, 1 N Grand Blvd, St. Louis, MO 63103, USA; travis.loux@slu.edu; 4 College of Nursing and Health Sciences, Texas A&M University, 5201 University Blvd, Laredo, TX 78041, USA; levi.ross@tamiu.edu

**Keywords:** ASD, ADHD, neurodevelopmental disorders, Medicaid expansion

## Abstract

Background/Objectives: Children with Autism Spectrum Disorder (ASD) and Attention-Deficit/Hyperactivity Disorder (ADHD) frequently rely on pharmacological treatment to manage core symptoms. This study examined how Medicaid expansion and the COVID-19 pandemic influenced medication use among children with ASD or ADHD, including those with comorbid diagnoses. Methods: We analyzed 2016–2023 data from the National Survey of Children’s Health (NSCH) for children aged 3–17 years with caregiver-reported diagnoses. Logistic regression models assessed the association between Medicaid expansion, the pandemic period, and current medication use, including an interaction between expansion and pandemic period. Analyses were conducted for the full sample (N = 35,198) and a subgroup with comorbid ASD and ADHD (N = 4298). Results: Current Medicaid expansion was associated with significantly lower odds of medication use in the full sample (aOR = 0.68, *p* < 0.001) but not the comorbid group (aOR = 0.98, *p* = 0.9). Medication use showed no significant change during the COVID-19 period in either the full sample (aOR = 0.99; *p* > 0.90) or the comorbid subgroup (aOR = 1.22; *p* = 0.4). A significant interaction indicating increased odds of medication use during the pandemic in expansion states was observed only in the full sample, although a similar but non-significant pattern appeared in the comorbid group. Age, race, and insurance-related differences were significant across groups, with coverage consistency playing a larger role in the full sample. Sensitivity analyses, excluding the 2020 survey year and modeling pre/post pandemic periods, supported the robustness of findings. Conclusions: Medicaid expansion was associated with patterns consistent with buffering pandemic-related disruptions in medication use among children with ASD or ADHD overall, but those with co-occurring conditions remain especially vulnerable.

## 1. Introduction

Attention-Deficit/Hyperactivity Disorder (ADHD) is typically marked by persistent patterns of inattention, hyperactivity, and impulsivity that interfere with functioning across academic, social, and familial settings [1]. Autism Spectrum Disorder (ASD), in contrast, is characterized by difficulties in social communication alongside restricted and repetitive behaviors [2]. Although these conditions are diagnostically distinct, considerable research highlights a meaningful overlap in symptoms. Children with ASD frequently display hyperactivity and inattention, which are hallmark traits of ADHD, while individuals with ADHD may show social communication deficits and behavioral patterns more commonly associated with ASD [3]. These shared characteristics have led researchers to posit common genetic and neurodevelopmental pathways underlying both disorders [4,5]. Comorbidity is also frequent, with studies showing that many children simultaneously meet the criteria for both diagnoses [6,7,8,9].

The chronic nature of both disorders further compounds this burden by necessitating long-term care, specialized support services, and educational accommodations [10]. Pharmacological treatment is a well-established first-line approach for ADHD, with stimulant medications like methylphenidate demonstrating high efficacy and large effect sizes [11,12,13]. In contrast, treatment for ASD often emphasizes behavioral interventions, with medication reserved for co-occurring ADHD symptoms such as irritability or hyperactivity [14]. Central nervous system stimulants such as methylphenidate have been shown to produce the largest and quickest reduction in ADHD symptoms [15,16,17], and are especially common in cases where ADHD co-occurs with ASD [18,19]. These medications are known to reduce core ADHD symptoms in approximately 80% of affected children and adolescents [15,20,21]. When prescribed, they often lead to improvements in attention span, task completion, classroom productivity, and behaviors like note-taking and handwriting [22,23,24]. Access to these medications is typically widespread through primary care settings, increasing their usage across diverse populations [25,26]. Approximately 27% of children with ASD are prescribed medication to manage related symptoms, with higher rates among those who have behavioral issues [27,28]. In 2007 to 2008, an estimated 4.8% of U.S. children aged 4 to 17, or about 2.7 million, were receiving ADHD medications [29]. By 2017, this figure had risen to 5.2% [30]. Nearly half of children with both ASD and ADHD use psychotropic medication, highlighting the complexity of co-occurring diagnoses [28]. Insurance coverage shapes access to treatment, whereby children covered by Medicaid (public insurance) were reportedly more likely to receive medication for ADHD or ASD compared to those with private insurance [31,32,33].

Caring for children with ADHD or ASD often comes with significant financial strain. Healthcare costs for families managing these conditions tend to be higher, particularly for those with limited income or fewer resources [34,35,36]. For low-income households, these burdens are compounded, as children with ADHD or ASD often have elevated healthcare needs due to high rates of comorbidity and lifetime prevalence [37,38,39,40], while also facing systemic barriers that limit their access to adequate care [36,41]. This financial exposure is consistent with broader evidence that health shocks and medical spending risk can reshape household financial behavior and debt trajectories [42,43], and is heightened during macroeconomic crises that amplify debt [44]. In response to systemic healthcare inequities, the Affordable Care Act (ACA) introduced sweeping reforms that improved access to medical and mental health services for children with neurodevelopmental disorders (NDDs) [45]. It prohibited insurers from denying coverage due to preexisting conditions and required parity between mental and physical health services, recognizing behavioral healthcare as essential [45,46,47,48,49]. The ACA also mandated preventive services for children, including mental health screenings and developmental assessments, supporting early identification and intervention [50]. It promoted integrated care models, such as medical homes and collaborative care, which expanded access to multidisciplinary services for children with NDDs [51]. To further improve affordability, the ACA expanded Medicaid and shifted many low-income children from the Children’s Health Insurance Program (CHIP) to Medicaid for families earning below 138% of the Federal Poverty Level [52]. These changes collectively increased insurance coverage and reduced out-of-pocket costs for low-income families [53,54]. However, states were granted flexibility in administering CHIP through expansion, standalone, or hybrid models [55].

The expansion of healthcare access through the ACA and CHIP set the stage for evaluating the effects of these policies during the COVID-19 pandemic. Evidence also suggests that both insurance design and recession-era policy responses can shape household debt, healthcare use, and financial stability following shocks [43,56,57]. Several studies have examined how Medicaid expansion influenced pandemic-related health outcomes across states [58,59,60,61,62,63,64,65], with expansion states found to have about 217 fewer COVID-19 cases per 100,000 residents than non-expansion states. Similarly, Gee [66] argued that Medicaid expansion improved states’ capacity to respond to the pandemic. Apenyo et al. [58] noted no major differences in infection rates based on expansion status but found disparities in mortality by income level. Furthermore, job losses in expansion states were reportedly less likely to result in loss of insurance coverage due to broader Medicaid eligibility [61]. These pandemic-era patterns align with evidence that Medicaid expansion can function as a financial buffer by improving insurance continuity and limiting unpaid medical bills during employment shocks [67,68].

Despite these findings, children with NDDs such as ASD and ADHD remain underexamined in this context. While some research has addressed the pandemic’s effects on these children [69,70,71,72,73,74], none have assessed differences by Medicaid expansion status. Previous studies on vulnerable groups have largely focused on adults with chronic conditions or socio-economic disadvantages, leaving children with ASD or ADHD notably absent from the Medicaid expansion literature [75,76,77]. This omission is notable, given evidence that the financial protection conferred by insurance can be heterogeneous across populations [42]. Even under Medicaid expansion, some groups continue to report medical debt and cost avoidance, suggesting limits to coverage-based protection in high-need or structurally disadvantaged settings [78], reinforcing the importance of examining how policy context corresponds with treatment patterns rather than assuming uniform gains. In light of this, the limited focus on children with ASD or ADHD in Medicaid expansion research, this study addresses a key gap by examining whether pharmacological treatment use differs between children in expansion and non-expansion states. It also explores whether such differences in access and treatment continued during the COVID-19 pandemic. Building on findings that Medicaid expansion improved healthcare access and reduced coverage loss during the crisis, this research investigates whether those gains extended to children with NDDs and whether this was accompanied by differences in pharmacological treatment or potentially different care patterns. Ultimately, the study aims to assess how state-level policy context may be related to the continuity and equity of pharmacological care before and after the pandemic, with a specific focus on whether the patterns observed also hold among children with dual ASD and ADHD comorbidity.

## 2. Materials and Methods

We conducted a repeated cross-sectional analysis using pooled data from the 2016–2023 waves of the National Survey of Children’s Health (NSCH), a nationally representative, caregiver-reported survey administered annually by the U.S. Census Bureau and collected from households in all 50 states and Washington, DC [79,80]. The NSCH provides national cross-sectional estimates on child health, service access, and social determinants of health for U.S children under the age of 18 [81]. The analysis began in 2016 to ensure consistency across years following a major survey redesign that introduced expanded behavioral health content and shifted data collection to mail and web-based formats [82,83].

Analytical data were restricted to responses of the survey questions: “Has a doctor or other health care provider EVER told you that this child has ADD or ADHD” and “Has a doctor or other health care provider EVER told you that this child has Asperger’s syndrome or autism.” Our analytic sample included children aged 3–17 years whose caregivers reported a current diagnosis of ADHD, ASD, or both conditions (N = 35,196). By design, children without a current diagnosis of either disorder (N = 244,110) were excluded, as the study focuses specifically on service utilization among children actively living with one or both of these neurodevelopmental disorders. The primary outcome was caregiver-reported current use of prescription medication for ADHD or ASD-related symptoms, including autism, Asperger’s disorder, pervasive developmental disorder (PDD), or attention-deficit disorder (ADD).

The primary exposures were the state’s Medicaid expansion status and the COVID-19 time period. Medicaid expansion status was treated as a time-varying, state-level policy variable based on year of implementation under the ACA. States were coded as “non-expansion” before implementation and “expansion” thereafter. States were considered non-expansion in the year they implemented the policy and expansion in all subsequent years. States that had not expanded Medicaid by 2023 (e.g., Texas, Florida, South Dakota) were classified as non-expansion throughout the study period. A detailed breakdown of state classifications by year is presented in Table 1. The COVID-19 period was dichotomized as pre-pandemic (2016–2019) and during/post-pandemic (2020–2023).

We first generated descriptive statistics to compare participant characteristics across the four policy-time groups, using design-adjusted chi-square tests for differences. Multivariable logistic regression models were then estimated to examine the association between Medicaid expansion, the COVID-19 period, and their interaction with current medication use. A stepwise model-building strategy was used: Model 1 included main effects, while Model 2 introduced an interaction term between Medicaid expansion and the COVID-19 period. The interaction reflects the differential change in current medication use in expansion states compared to non-expansion states following the onset of the pandemic. All models included covariates selected a priori based on prior literature and conceptual relevance to medication use among children with ASD or ADHD [84,85,86,87,88,89,90,91,92,93]. These included child age group (3–5, 6–11, 12–17), sex, race and ethnicity, primary language spoken in the household, family structure, and metropolitan versus non-metropolitan residence. Socioeconomic variables included the federal poverty level category and the highest level of education among adults in the household. Insurance-related variables included current insurance type (public, private, dual, or uninsured), insurance coverage consistency over the prior 12 months, perceived adequacy of the child’s coverage in meeting health needs, and reported difficulty paying the child’s medical or health care bills in the past year.

These analyses were first conducted for the full analytic sample (N = 35,196). The same modeling strategy was then replicated in a planned subgroup analysis restricted to children with co-occurring ASD and ADHD (N = 4298) to assess whether predictors of medication use differed in this higher-needs population.

All models were estimated using R version 4.5.1 and incorporated the NSCH’s complex survey design, applying the provided strata, clusters by state, and survey weights to produce nationally representative estimates. Results are reported as adjusted odds ratios (aORs) with 95% confidence intervals (CIs), with statistical significance defined as *p* ≤ 0.05. A sensitivity analysis was also performed, excluding the 2020 survey year to account for potential measurement disruptions during the early stages of the pandemic.

## 3. Results

The analytic sample comprised 35,196 children aged 3 to 17 years whose caregivers reported a diagnosis of ADHD, ASD, or both. Of these, 30,898 children (87.8%) had a diagnosis of either ASD or ADHD, while 4498 (12.2%) had been diagnosed with both conditions. Notably, the distribution of Medicaid expansion residence and survey timing was similar across diagnostic groups. Among children with either condition, 20.6% resided in Medicaid expansion states, and 79.4% in non-expansion states; for those with both conditions, 20.4% lived in Medicaid expansion states and 79.6% in non-expansion states. Likewise, 38.1% of children with either condition and 34.8% of those with both were surveyed before the COVID-19 pandemic (2016–2019), while 61.9% and 65.2%, respectively, were surveyed during or after the pandemic period (2020–2023). Table 2 presents the distribution of current medication use, survey timing, and Medicaid expansion status by diagnostic group. Table S1 and Table S2 present the remaining descriptive characteristics of participants by Medicaid expansion and the COVID-19 period by diagnostic group, respectively.

### 3.1. Medication Use by Diagnostic Profile

Medication use among children with at least one diagnosis (ASD or ADHD) was relatively balanced, with 16,239 children (52.6%) currently using prescription medication and 14,659 (47.4%) not receiving medication. Among the subgroup of children diagnosed with both ASD and ADHD, however, medication use was less common. Only 1630 children in the comorbid subgroup (36.2%) were currently taking medication, while 2668 children (63.8%) were not. This pattern suggests that children with co-occurring ASD and ADHD may face more barriers or different clinical considerations related to pharmacological treatment.

### 3.2. Multivariable Regression Results

Survey-weighted logistic regression results are presented below, estimating the associations between Medicaid expansion, the COVID-19 period, and current medication use. Summary results of the models are shown in Table 3, with full model results in Tables S3 and S4.

#### 3.2.1. Medicaid Expansion

In Model 1, the Medicaid expansion period was associated with significantly lower odds of current medication use in the full analytic sample (aOR = 0.68; 95% CI = 0.60–0.77; *p* < 0.001). In contrast, the expansion period had a minimal and not statistically significant association with medication use in the ASD + ADHD subgroup (aOR = 0.98; 95% CI = 0.74–1.30; *p* > 0.90), suggesting that expansion status was not a consistent predictor of medication use in this higher-needs population.

#### 3.2.2. COVID-19 Effect

The COVID-19 period had almost no association with the odds of medication use in Model 1. Among children surveyed during or after the pandemic, the odds of medication use were similar to those surveyed before in the full sample (aOR = 0.99; 95% CI = 0.79–1.25; *p* > 0.9) and a higher but still not statistically significant in the ASD + ADHD subgroup (aOR = 1.22; 95% CI = 0.73–2.05; *p* = 0.4).

#### 3.2.3. Medicaid Expansion × COVID-19 Interaction

In Model 2, the interaction between Medicaid expansion and the COVID-19 period was statistically significant in the full sample (aOR = 1.35; 95% CI = 1.05–1.74; *p* = 0.020) but not the ASD + ADHD subgroup (aOR = 1.60; 95% CI = 0.90–2.86; *p* = 0.11). The direction of the interaction was positive in both groups, indicating a potential moderating relationship between Medicaid expansion and the pandemic in describing medication use.

#### 3.2.4. Sociodemographic and Insurance Characteristics

In addition to the primary associations of Medicaid expansion and the COVID-19 pandemic, the interaction models produced additional significant associations (Tables S3 and S4). Age was the most consistent predictor of medication use. Compared to children aged 3 to 5, those aged 6 to 11 and those aged 12 or older had significantly higher odds of medication use in both the full analytic sample (aORs = 5.36 and 5.13; *p* < 0.001) and the ASD plus ADHD subgroup (aORs = 5.41 and 5.16; *p* = 0.003). Children living in non-metropolitan areas also had higher odds of medication use (aOR = 1.15; *p* = 0.047). These findings suggest that age and geographic location were key predictors of medication use regardless of diagnostic subgroup.

Children from non-English-speaking households had lower odds of medication use (aOR = 0.59; *p* < 0.001). Similarly, non-Hispanic children had higher odds of medication use than Hispanic children in the full sample (White: aOR = 1.63; Black: aOR = 1.38; multi-racial: aOR = 1.21; *p* < 0.001), but these differences were not significant in the subgroup. Family structure was also predictive in the full sample, where living with two unmarried parents was associated with lower odds (aOR = 0.74; *p* < 0.001) and living with grandparents or other relatives was associated with higher odds (aOR = 1.34; *p* < 0.001); these associations were not observed in the subgroup. Insurance-related predictors followed a similar pattern. Inconsistent coverage (aOR = 0.63; *p* = 0.007) had lower odds of medication use in the full sample, but this association was not significant in the subgroup. Likewise, insurance type was significant in the comorbid group but not in the full sample. Sex, perceived adequacy of coverage, and difficulty paying for care were not significantly associated with medication use in either group.

### 3.3. Imputation

Before imputation, we assessed the extent of missingness for all analytic variables. Most variables had minimal missing data (<2%). Specifically, child age, sex, and race/ethnicity were fully observed. Missingness was higher for socioeconomic indicators: metropolitan residence had the highest proportion of missing data at 19.5%, family income as a percentage of the federal poverty level (FPL) was missing in 4.7% of cases, highest household educational attainment in 3.9%, and insurance continuity in 2.5%. The adequacy of insurance coverage and difficulty paying medical bills variables had missingness rates of 1.6% and 2.2%, respectively. To minimize potential bias, we performed single-value imputation: median imputation was used for ordinal and continuous variables, while mode imputation was applied to categorical variables. After imputation, we verified data completeness using summary statistics and ensured there were no remaining missing values in the analytic sample. This approach aligns with established practices for handling low-to-moderate levels of missingness in complex survey datasets like the NSCH.

### 3.4. Sensitivity Analysis

As a sensitivity analysis to assess the robustness of the main findings, data from the 2020 survey were removed due to the potential for mismeasurement caused by pandemic disruptions. Results from the sensitivity analysis were broadly similar to those of the main analysis. Full model results are available in Table S5, along with a figure (Figure S1) comparing the primary estimates of interest across all three analyses. These results support the robustness of the main findings and suggest that Medicaid expansion may have helped buffer medication continuity during the pandemic.

## 4. Discussion

This study examined how state-level Medicaid expansion policy was related to the continuity and equity of pharmacological care for children diagnosed with ASD and/or ADHD in the period before and after the COVID-19 pandemic. While the primary analysis focused on the overall population, additional subgroup analysis was conducted to assess whether observed patterns similarly applied to children with comorbid ASD and ADHD. While the effects of the COVID-19 period and demographic factors like age group were directionally consistent across both the full sample and the morbid subgroup, key associations related to Medication expansion and medication use differed. These mixed patterns suggest that structural policies and clinical decision-making may be associated with different treatment patterns depending on diagnosis complexity.

### 4.1. Medication Use Patterns

Although the primary analysis was not designed to compare children with a single diagnosis to those with comorbidity, the subgroup analysis focusing on children with ASD and ADHD provides valuable insight into medication use in this population. Prior research consistently demonstrates that comorbidity between ASD and ADHD is common and clinically consequential [94,95]. Studies estimate that 40% to 70% of children with ASD meet criteria for ADHD, and 30% to 75% of children and youth with ADHD exhibit ASD traits [12,96,97,98,99]. This overlap is associated with more severe impairments in cognition, behavior, attention, inhibition, and adaptive functioning [20,100,101,102,103,104]. Treatment becomes more complex in children with comorbid ASD and ADHD. They are also more likely to display externalizing symptoms, have a lower quality of life, and respond less effectively to behavioral interventions such as social skills training [101,105,106]. Additionally, diagnostic clarity is more difficult to achieve when both conditions are present, as overlapping symptoms can obscure clinical presentation [7], whereas children with both conditions may be less likely to receive evidence-based intervention for ADHD symptoms than those with ADHD alone [97]. Although atomoxetine, guanfacine, and stimulants have shown benefits in this group [107,108,109], prescribing tends to be more cautious, influenced by historical concerns about tolerability. Prior studies reported increased side effects in children with ASD, such as irritability and social withdrawal [110,111], though recent evidence suggests comparable efficacy and safety profiles between ASD + ADHD and ADHD-only groups [12,112]. These lingering perceptions, along with the need for individualized care plans and slower titration, may contribute to patterns of lower or delayed medication use among children with comorbid presentations [97,112,113].

The literature on medication use in this group remains mixed. Frazier et al. [114], using data from adolescents aged 13 to 17 in special education programs collected between 2000 and 2009, found higher rates of psychotropic medication use among youth with ASD and ADHD than among those with either condition alone. In the multisite clinical registry, higher psychotropic medication use was reported in children with ASD + ADHD (58.2%) compared to those with ADHD alone (49.0%) or ASD alone (34.3%). Rast et al. [28], analyzing 2016–2017 NSCH data, reported that children with comorbid ASD and ADHD had comparable or slightly higher medication use than those with ADHD alone. In contrast, Zablotsky et al. [102], using 2014 national survey data, found that children with both diagnoses had greater treatment needs, more co-occurring conditions, and were more likely to present with a combined ADHD subtype, though the study did not assess actual medication use. These findings point to a broader uncertainty in the field regarding how clinical complexity affects pharmacological care. The present study contributes new insight by documenting lower rates of medication use among children with comorbid ASD and ADHD using a more recent and nationally representative sample spanning 2016 to 2023. This extended timeframe captures evolving treatment trends during and after the COVID-19 pandemic and provides a more current and comprehensive view of pharmacological use across diagnostic groups. When considered alongside earlier findings of heightened clinical need, the lower treatment rates observed in our study are consistent with a potential disconnect between the complexity of care needs and access to consistent pharmacological treatment.

Taken together, these findings raise important questions about whether current clinical and systemic practices adequately meet the needs of children with comorbid ASD and ADHD. The lower treatment rates observed may reflect ongoing provider hesitation, fragmented service delivery, and a lack of standardized, diagnosis-sensitive treatment pathways. Addressing these disparities will require improved clinical guidance, targeted provider training, and integrated care models capable of coordinating behavioral and pharmacological approaches for children with dual diagnoses.

### 4.2. Medicaid Expansion

To our knowledge, this study is the first to examine the association between Medicaid expansion and pharmacological treatment among children with ASD, ADHD, or both. Prior studies of adult populations and general prescription use consistently reported increases in medication utilization and reimbursement in expansion settings, including a 17.0% increase in prescriptions and a 36.1% rise in reimbursement [115]. Similar increases in access have been documented for diabetes medications [116], psychotropic treatments [117], and naloxone dispensing for opioid overdose [118]. Prior research also suggests that expansion corresponds with greater service availability for vulnerable populations [119]. However, these studies did not specifically evaluate pediatric neurodevelopmental diagnoses. In contrast, expansion status was associated with reduced medication use in our study population. Notably, this inverse relationship was only statistically significant among children with either ASD or ADHD alone, but non-significant among those with a comorbid diagnosis. Together, these results indicate a different pattern of policy-treatment relationship in pediatric healthcare.

Several system-level differences between expansion and non-expansion states may help explain why medication use was lower in expansion states in this study. Expansion states have seen growth in behavioral health infrastructure, including a 9% increase in Board Certified Behavioral Analysts (BCBA) and a 5% increase in child psychiatrists [120]. Additional studies have documented broader access to preventive and primary care in expansion states [121,122,123], improved care continuity [124], and increased Medicaid enrollment [125]. These developments may coincide with greater access to non-pharmacologic services and reduced reliance on medication, particularly for children with less complex needs. This may be particularly relevant in the context of Medicaid’s Early and Periodic Screening, Diagnostic, and Treatment (EPSDT) benefit, which requires states to provide all medically necessary services to children, including behavioral health interventions [126]. In this context, reduced reliance on medication may reflect a shift toward earlier or more comprehensive use of behavioral services, rather than reduced access to care.

Overall, these findings indicate that Medicaid expansion does not correspond to a uniform increase in medication use among children with NDDs. Instead, the observed relationships vary by diagnostic complexity and the broader care environment, reflecting how policy intersects with service infrastructure and clinical practice patterns. Future work should investigate these dynamics more closely, particularly the balance between pharmacological and behavioral health services in expansion versus non-expansion states.

### 4.3. COVID-19 Impact

In this study, medication use was lower during and after the COVID-19 period in the full analytical sample, whereas the comorbid ASD and ADHD group showed higher odds of medication use. However, neither estimate reached statistical significance, indicating that the observed differences were modest and uncertain. Despite this lack of significance, the direction of the association in the full sample is consistent with a growing body of evidence documenting disruptions to pediatric pharmacological treatment during the COVID-19 pandemic. The rapid expansion of telehealth services helped support continuity of care during the pandemic [127,128]. In addition, Medicaid enrollment reached historic highs due to policies that preserved eligibility and limited disenrollment [129]. Even so, the overall pattern in our results is consistent with the possibility that these measures were not sufficient to prevent declines in medication use and that broader system-level disruptions may have outweighed these protective efforts.

In a U.S.-based study, Mirza et al. [130] reported a substantial decline in ADHD prescriptions among youth aged 10–19 during full school closures, followed by a gradual recovery as schools reopened. National trends further show substantial reductions in pediatric prescriptions during 2020, when total dispensed medications declined by 27% (April–December 2020 vs. 2019), and ADHD medication dispensing dropped by 11.8% [131]. Similar declines were documented during complete school closures, when ADHD medication use among youth fell sharply [130]. Cunniff et al. [132] similarly found that ADHD medication fill rates dropped from 40 to 66% pre-pandemic to 31–44% during pandemic months, despite refill continuity and adherence being highest among patients who received both virtual and in-person visits [132].

International evidence points to similar patterns of disruptions. Sciberras et al. [133], in an Australian survey, reported that 16% of children with ADHD had stopped taking medication, largely due to school closures and routine disruption. In Egypt, Yousef et al. [134] found that over 74% of children with ADHD were non-adherent to medication during the pandemic. These international findings suggest that declines in medication use were not unique to the United States but may reflect global patterns in pediatric behavioral health treatment disruptions.

Notably, no existing study to our knowledge has directly examined the pandemic’s impact on medication use among children with comorbid ASD and ADHD. Although our findings do not provide conclusive evidence of a pandemic-related change in this high-need subgroup, the opposing direction of the estimate suggests that pandemic-era shifts in service delivery may have been related to medication use differently for children with dual diagnosis than for the broader population. These results underscore the need for further research with greater statistical power to clarify how pandemic-era changes in service delivery influenced pharmacological treatment for children with complex neurodevelopmental conditions.

### 4.4. Medicaid Expansion × COVID-19 Interaction

Although point estimates were directionally positive in both the full sample and among children with co-occurring ASD and ADHD, indicating higher odds of medication use in expansion states during the pandemic, the interaction reached statistical significance only in the full sample. This pattern is consistent with the possibility that Medicaid expansion was associated with more stable pharmacological treatment during and after the pandemic for children with ASD or ADHD more broadly. One possible explanation is the role of continuous coverage protections enacted during the public health emergency, which contributed to record-high Medicaid and CHIP enrollment [129]. These protections, along with the eligibility and enrollment infrastructure improvements documented in expansion states [121,122,123,124,125], may have been related to more sustained treatment access in expansion states during a period of widespread healthcare disruption [135]. Several other mechanisms may help contextualize this interaction, though they were not directly tested in the present analysis. Compared to non-expansion states, expansion states experienced earlier rebounds in full-year Medicaid coverage during the pandemic [136], and uninsurance rates modestly decreased in expansion states after the onset of COVID-19, while remaining stable or worsening in non-expansion states [137].

The absence of statistically significant effects in the comorbid subgroup should not be interpreted as a lack of policy relevance, however. It suggests that coverage continuity alone may not have been sufficient to preserve access to medication in the face of more complex clinical, educational, or caregiving needs. As previously noted, children with dual diagnoses often present with greater clinical complexity and more heterogeneous care needs, which may weaken the observable associations of state-level policies relative to individual-level clinical decision-making. These trends highlight the need for future studies with larger and more targeted samples to better understand how policy and clinical factors interact to shape care for children with co-occurring neurodevelopmental conditions.

Nonetheless, these findings should also be interpreted in the context of post-pandemic changes to Medicaid enrollment. After continuous coverage protections ended in April 2023, the nationwide unwinding led to disenrollment of more than four million children by the end of that year [138]. Losses were disproportionately concentrated in large non-expansion states, including more than one million children in Texas [138]. While the present results suggest that Medicaid expansion was associated with higher odds of pharmacological treatment during and after the pandemic for children with ASD or ADHD, these gains may not persist without continued structural protections [139,140]. Ensuring long-term treatment continuity for children with neurodevelopmental conditions will require stable coverage and sustained investment in pediatric health services.

### 4.5. Sociodemographic and Household Characteristics

This study reinforces previous findings that age is a strong and consistent determinant of medication use among children with ASD and ADHD. In both diagnostic groups, school-aged and adolescent children were significantly more likely to receive medication compared to preschool-aged children, with more pronounced differences observed in the broader analytic sample. These patterns are consistent with prior research showing increased psychotropic use as children grow older, likely reflecting greater diagnostic clarity, rising behavioral demands, and evolving clinical priorities during later developmental stages [28,33,141,142,143]. While age-related increases were also observed among children with co-occurring ASD and ADHD, the difference across age groups was less marked, suggesting that treatment patterns in this subgroup may be influenced by additional factors beyond age alone. Together, these findings highlight the importance of age-specific approaches to pharmacological treatment and the need for careful monitoring as children with neurodevelopmental conditions mature.

Other sociodemographic characteristics also influenced medication use, although associations varied by diagnostic group. Consistent with earlier research, race, language spoken at home, and household structure were significantly associated with treatment in the full sample [33,141]. However, these associations did not persist within the comorbid ASD + ADHD subgroup, suggesting both limited sample size and the greater heterogeneity of treatment pathways among children with dual diagnoses [144,145,146,147]. Medication use was lower among children with private insurance only, or inconsistent coverage, in both diagnostic groups, but the strength and significance of these associations differed. In the full sample, both factors were significantly associated with reduced use, aligning with prior studies on access disparities that have been linked to insurance type and continuity of care [148,149]. In the comorbid group, only the association with private insurance remained significant, suggesting that while insurance type still influences access, the effects of coverage gaps may be less pronounced in children with more complex needs [150].

These contrasts between the full sample and the ASD + ADHD subgroup imply that the influence of sociodemographic and insurance factors on treatment may be less pronounced or operate differently when clinical complexity increases. Understanding these nuances is important for tailoring interventions and ensuring equitable access to care, as children with dual diagnoses may require different strategies to engage in treatment compared to children with a single diagnosis.

### 4.6. Limitations

This study has several limitations. First, the repeated cross-sectional design limits causal inference. Although Medicaid expansion status was modeled as a time-varying state policy exposure and the COVID-19 period was operationalized as a pre- versus during/post-pandemic indicator with an interaction term, the data cannot establish temporal order at the individual level or capture within-child changes over time. Consequently, these analyses identify associations rather than definitive causal effects, and residual confounding remains possible in observational policy analyses even with covariate adjustment [151,152,153,154]. Longitudinal designs would better support evaluation of within-person trajectories in service use and medication continuity before and after policy and period changes [154].

Second, reliance on parent-reported measures may introduce measurement error and bias, and unmeasured confounding remains possible. Caregivers reported their diagnostic status and current medication use, but these reports were not validated through clinical assessment, pharmacy claims, or medical records, consistent with the NSCH’s survey-based design [79,80,82,83]. Parent-reported provider diagnosis and medication use are widely used in national surveillance for ASD and ADHD, but they remain subject to misclassification, recall error, misunderstanding of diagnostic terms, and social desirability effects [27,29,155,156,157]. Misclassification and reporting error may also vary across populations and contexts, which could contribute to differential measurement error across states or time periods [153,156]. In addition, medication use was measured as a binary indicator, limiting the ability to assess dosage, adherence, duration, medication class, polypharmacy, switching, or treatment indication, which constrains interpretation of treatment intensity and purpose [156,158]. Beyond measurement issues, residual confounding is possible because the models could not fully capture state and local differences in service availability, practice patterns, and contemporaneous policy and administrative changes that may correlate with both expansion status and medication use [75,149]. This concern is particularly salient during the pandemic period, when Medicaid enrollment, financing, and coverage continuity were shaped by pandemic-era provisions and subsequent unwinding processes that varied across states and over time, potentially affecting access and utilization in ways not fully captured by expansion status alone [129,135,138,139]. Furthermore, the comorbid ASD and ADHD subgroup was smaller than the full sample. This limited sample size likely reduced the statistical power to detect significant associations in the subgroup models, even when effect directions were consistent with the main analysis. With a larger sample, it is plausible that future studies would find statistically significant associations in both diagnostic groups. Larger, pooled, or longitudinal datasets may therefore provide a more definitive assessment of subgroup effects.

Finally, although multiple imputation was used to address missing data, this approach assumes data are missing at random conditional on observed variables and may not fully correct for systematic differences between respondents with complete and incomplete data [159]. Future research should use longitudinal designs and, where feasible, linked clinical or claims data to validate caregiver-reported diagnoses and medication use, distinguish initiation from continuation, incorporate measures of medication class and intensity, and better characterize policy and system heterogeneity to strengthen inference and generalizability.

### 4.7. Clinical Practice Implications

Children with co-occurring ASD and ADHD had lower rates of medication use compared to the broader neurodevelopmental group, even after adjusting for sociodemographic and insurance-related factors. This highlights the need for more tailored treatment approaches that account for the complexity of comorbid presentations. The lack of a significant association between Medicaid expansion status and medication use in this subgroup suggests that insurance coverage alone may not be sufficient to ensure access to appropriate pharmacologic care. Additionally, declines in medication use during the pandemic, despite expanded telehealth and high enrollment in public insurance, point to broader vulnerabilities in care continuity. Clinicians managing children with ASD or ADHD should be supported through training, decision-making tools, and clearer treatment guidelines, particularly when addressing overlapping symptomatology. Strengthening collaborative care models may help coordinate behavioral and medical treatment, improving outcomes for children with high service needs.

## 5. Conclusions

This study found that Medicaid expansion was associated with lower overall odds of medication use among children with ASD or ADHD. At the same time, during the COVID-19 pandemic, children in expansion states showed relatively higher medication use compared with those in non-expansion states, suggesting that expansion may have moderated pandemic-related declines in the broader population. This pattern did not extend to children with co-occurring ASD and ADHD, for whom expansion was not associated with a meaningful difference in medication use during the pandemic. Together, these findings indicate that the relationships between Medicaid expansion and pharmacologic treatment are context-dependent and vary by diagnostic complexity. While expansion may be related to differences in treatment patterns under stable conditions and during system-wide disruptions, it does not appear to operate uniformly across subgroups with higher clinical needs. However, these findings should be interpreted within the context of the repeated cross-sectional design of the study and its reliance on caregiver-reported measures, which provide limited information on several medication characteristics and may not fully account for unmeasured state-level or pandemic-era influences, particularly for the smaller comorbid subgroup. Future research should use longitudinal, larger-scale datasets and linked clinical or claims records to improve causal inference, validate outcomes, and better characterize treatment patterns across subgroups and time.

## Figures and Tables

**Table 1:** State Medicaid Expansion Status.

State	Pre-COVID-19				During or Post-COVID-19			
	2016	2017	2018	2019	2020	2021	2022	2023
Alabama	Non-Expansion							
Alaska	Expansion							
Arizona	Expansion							
Arkansas	Expansion							
California	Expansion							
Colorado	Expansion							
Connecticut	Expansion							
Delaware	Expansion							
Washington DC	Expansion							
Florida	Non-Expansion							
Georgia	Non-Expansion							
Hawaii	Expansion							
Idaho ^1^	Non-Expansion					Expansion		
Illinois	Expansion							
Indiana	Expansion							
Iowa	Expansion							
Kentucky	Non-Expansion							
Louisiana ^2^	NE	Expansion						
Maine ^3^	Non-Expansion				Expansion			
Maryland	Expansion							
Massachusetts	Expansion							
Michigan	Expansion							
Minnesota	Expansion							
Mississippi	Non-Expansion							
Missouri ^4^	Non-Expansion						Expansion	
Montana ^5^	NE	Expansion						
Nebraska ^6^	Non-Expansion				Expansion			
Nevada	Expansion							
New Hampshire	Expansion							
New Jersey	Expansion							
New Mexico	Expansion							
New York	Expansion							
North Carolina	Non-Expansion							
North Dakota	Expansion							
Ohio	Expansion							
Oklahoma ^7^	Non-Expansion					Expansion		
Oregon	Expansion							
Pennsylvania	Expansion							
Rhode Island	Expansion							
South Carolina	Non-Expansion							
South Dakota	Non-Expansion							
Tennessee	Non-Expansion							
Texas	Non-Expansion							
Utah ^8^	Non-Expansion						Expansion	
Vermont	Expansion							
Virginia ^9^	Non-Expansion				Expansion			
Washington	Expansion							
West Virginia	Expansion							
Wisconsin	Non-Expansion							
Wyoming	Non-Expansion							

^1^ Idaho adopted Medicaid Expansion in 2018 but began implementation in January 2020. ^2^ Louisiana adopted and implemented Medicaid Expansion in 2016. ^3^ Maine adopted Medicaid Expansion in 2017 but began implementation in January 2019 with coverage retroactive to July 2018. ^4^ Missouri adopted Medicaid Expansion in 2020 but began implementation in October 2021 with coverage retroactive to May 2021. ^5^ Montana adopted Medicaid Expansion in 2015 but began implementation in January 2016. ^6^ Nebraska adopted Medicaid Expansion in 2021 but began implementation in October 2020. ^7^ Oklahoma adopted Medicaid Expansion in 2020 but began implementation in June 2021, with coverage beginning in July 2021. ^8^ Utah adopted Medicaid Expansion in 2018 but began implementation in January 2020. ^9^ Virginia adopted Medicaid Expansion in 2018, but began implementation in January 2019. The information in this table was sourced from the Kaiser Family Foundation website (https://www.kff.org/affordable-care-act/state-indicator/state-activity-around-expanding-medicaid-under-the-affordable-care-act/?currentTimeframe=0&selectedRows=%7B%22states%22:%7B%22louisiana%22:%7B%7D,%22maine%22:%7B%7D,%22missouri%22:%7B%7D,%22montana%22:%7B%7D,%22nebraska%22:%7B%7D,%22oklahoma%22:%7B%7D,%22south-dakota%22:%7B%7D,%22utah%22:%7B%7D,%22virginia%22:%7B%7D%7D%7D&sortModel=%7B%22colId%22:%22Status%20of%20Medicaid%20Expansion%20Decision%22,%22sort%22:%22asc%22%7D, accessed on 20 July 2025).

**Table 2:** Analytical Sample Characteristics and Medication Use by Diagnostic Group (N = 35,196).

Diagnostic Group	Subgroup Characteristics	Count (% of Diagnostic Group)
At least one of ASD or ADHD	Currently on Medication	16,239 (52.6%)
	Not on Medication	14,669 (47.4%)
	Resides in Medicaid expansion state	6363 (20.6%)
	Resides in Medicaid non-expansion state	24,535 (79.4%)
	Surveyed Pre-COVID-19	11,784 (38.1%)
	Surveyed During/Post-COVID-19	19,114 (61.9%)
	Subtotal	30,898
Both ASD and ADHD	Currently on Medication	1630 (36.2%)
	Not on Medication	2668 (63.8%)
	Resides in Medicaid expansion state	876 (20.4%)
	Resides in Medicaid non-expansion state	3422 (79.6%)
	Surveyed Pre-COVID-19	1497 (34.8%)
	Surveyed During/Post-COVID-19	2801 (65.2%)
	Subtotal	4498

**Table 3:** Logistic Regression Analysis. Adjusted odds ratios, 95% confidence intervals, and *p*-values. All models adjusted for age, sex, race/ethnicity, language spoken at home, family structure, metro status, income relative to federal poverty level, highest level of household education, insurance type, insurance coverage consistency, insurance adequacy, and difficulty paying for child’s medical bills.

	ASD/ADHD		ASD + ADHD	
Model 1: Main effects	aOR (95% CI)	*p*-value	aOR (95% CI)	*p*-value
Current Expansion	0.68 (0.60, 0.77)	<0.001	0.98 (0.74, 1.30)	0.9
COVID Period	0.99 (0.79, 1.25)	>0.90	1.22 (0.73, 2.05)	0.4
Model 2: Interaction	aOR (95% CI)	*p*-value	aOR (95% CI)	*p*-value
Current Expansion	0.57 (0.46, 0.70)	<0.001	0.74 (0.46, 1.18)	0.2
COVID Period	0.84 (0.66, 1.08)	0.2	0.95 (0.53, 1.68)	0.8
Current Expansion × COVID-19 Period	1.35 (1.05, 1.74)	0.02	1.60 (0.90, 2.86)	0.11

## Data Availability

The original data presented in this study are openly available from the United States Census Bureau, https://www.census.gov/programs-surveys/nsch/data/datasets.html, accessed on 19 September 2024.

## Supplementary Materials

The following supporting information can be downloaded at: https://www.mdpi.com/article/10.3390/healthcare14050684/s1. Table S1: Descriptive Characteristics of Participants with Either ASD or ADHD, by Medicaid Expansion and COVID-19 Period; Table S2: Descriptive Characteristics of Participants with ASD and ADHD, by Medicaid Expansion and COVID-19 Period; Table S3: Full logistic regression results of the model for current medication use, main effects only; Table S4: Full logistic regression results of the model for current medication use, including the interaction between Medicaid expansion status and the COVID-19 period; Table S5: Full logistic regression results for sensitivity analysis, removing the 2020 year data collection; Figure S1: Logistic regression coefficients for Medicaid expansion, COVID-19 period, and their interaction in the full sample, comorbid subgroup, and sensitivity analysis.

## Abbreviations

The following abbreviations are used in this manuscript:

ACA	Affordable Care Act
aOR	Adjusted odds ratios
ADD	Attention Deficit Disorder
ADHD	Attention-Deficit/Hyperactivity Disorder
ASD	Autism Spectrum Disorder
BCBA	Board Certified Behavioral Analysts
CHIP	Children’s Health Insurance Program
CI	Confidence Interval
FPL	Federal Poverty Level
NDDs	Neurodevelopmental disorders
NE	Non expansion
NSCH	National Survey of Children’s Health
PDD	Pervasive Developmental Disorder
