# Supplementary material for: Medicaid Expansion and Medication Use Among U.S. Children with ASD or ADHD: A Repeated Cross-Sectional Analysis Comparing Before and During the COVID-19 Periods
Source: Healthcare (Basel). 2026 Mar 9;14(5):684. doi: 10.3390/healthcare14050684 (PMC12984698; doi:10.3390/healthcare14050684)
Supplement: Supplementary file 1 [file healthcare-14-00684-s001.zip › healthcare-4090491-supplementary.pdf]

Table S1: Descriptive Characteristics of Participants with Either ASD or ADHD, by Medicaid Expansion and COVID-19 Period

Variable	Expansion Status		COVID-19 Period		Chi2 (p-value)
	Expansion	Non-expansion	Pre-COVID-19	During/Post-COVID-19	
Age group					$\chi^2 = 238.75$ ($p < 0.001$)
Aged 3-5 years	170 (6.6%)	276 (10.7%)	459 (17.7%)	1,686 (65.1%)	
Aged 6-11 years	1,455 (11.1%)	1,290 (9.8%)	3,459 (26.4%)	6,918 (52.7%)	
Older than 12 years	2,295 (11.8%)	1,753 (9.0%)	5,443 (27.9%)	9,992 (51.3%)	
Race/Ethnicity					$\chi^2 = 169.70$ ($p < 0.001$)
Hispanic	396 (9.4%)	467 (11.1%)	985 (23.5%)	2,348 (56.0%)	
White, non-Hispanic	2,894 (11.7%)	2,219 (8.9%)	6,794 (27.3%)	12,944 (52.1%)	
Black, non-Hispanic	314 (12.7%)	327 (13.3%)	650 (26.4%)	2,466 (47.7%)	
Multi-racial, non-Hispanic	316 (8.6%)	306 (8.3%)	932 (25.3%)	3,683 (57.8%)	
Metro status					$\chi^2 = 90.45$ ($p < 0.001$)
Metropolitan	3,368 (11.2%)	2,763 (9.2%)	8,279 (27.5%)	15,741 (52.2%)	
Non-Metropolitan	552 (11.0%)	1,082 (11.0%)	2,855 (21.5%)	2,855 (56.6%)	
Family structure					$\chi^2 = 120.20$ ($p < 0.001$)
Two parents, currently married	2,483 (11.3%)	2,013 (9.2%)	5,851 (26.6%)	11,616 (52.9%)	
Two parents, not currently married	249 (11.1%)	170 (7.6%)	665 (29.6%)	1,165 (51.8%)	
Single parent (mother only)	826 (9.9%)	855 (10.3%)	2,034 (24.5%)	4,604 (55.3%)	
Grandparent household	362 (13.6%)	281 (10.5%)	811 (30.4%)	1,211 (45.4%)	

Education (Highest adult)					$\chi^2 = 10.50$ ($p = 0.11$)
< HS	93 (11.3%)	96 (11.6%)	224 (27.1%)	413 (50.0%)	
HS or GED	579 (10.6%)	546 (10.0%)	1,428 (26.2%)	2,900 (53.2%)	
Some college or more	3,248 (11.2%)	2,677 (9.3%)	7,709 (26.7%)	15,283 (52.9%)	
Insurance type					$\chi^2 = 108.04$ ($p < 0.001$)
Public only	1,147 (10.8%)	1,075 (10.1%)	2,666 (25.1%)	5,752 (54.1%)	
Private only	2,326 (11.4%)	1,903 (9.4%)	5,584 (27.4%)	10, 549 (51.8%)	
Public and private	272 (9.0%)	201 (6.7%)	798 (26.5%)	1,737 (57.8%)	
Uninsured	175 (14.8%)	140 (11.8%)	313 (26.4%)	558 (47.1%)	
Federal Poverty Level (FPL)					$\chi^2 = 72.91$ ($p < 0.001$)
0-199%	1,407 (11.7%)	1,227 (10.2%)	3,040 (25.4%)	6,307 (52.6%)	
200-299%	688 (11.6%)	602 (10.2%)	1,487 (25.1%)	3,140 (53.1%)	
300-399%	522 (11.3%)	441 (9.6%)	1,220 (26.5%)	2,419 (52.6%)	
400% or more	1,303 (10.3%)	1,049 (8.3%)	3,614 (28.5%)	6,730 (53.0%)	
Household language					$\chi^2 = 21.94$ ($p < 0.001$)
English	3,818 (11.2%)	3,206 (9.4%)	9,117 (26.8%)	17,941 (52.6%)	
Other	102 (9.2%)	113 (10.1%)	244 (21.9%)	655 (58.8%)	
Sex of child					$\chi^2 = 22.07$ ($p < 0.001$)
Male	2,717 (11.3%)	2,240 (9.3%)	6,580 (27.6%)	12,591 (52.2%)	
Female	1,203 (10.9%)	1,079 (9.8%)	2,781 (25.1%)	6,005 (54.3%)	
Insurance consistency					$\chi^2 = 43.34$ ($p < 0.001$)
Consistently insured	3,661 (11.0%)	3,111 (9.3%)	8,902 (26.6%)	17,763 (53.1%)	
Uninsured/inconsistent	259 (14.7%)	208 (11.8%)	459 (26.1%)	833 (47.4%)	
Insurance benefit coverage					$\chi^2 = 28.18$ ($p = 0.001$)
Always	2,055 (11.0%)	1,750 (9.4%)	5,049 (27.0%)	9,848 (52.7%)	
Usually	1,324 (11.0%)	1,104 (9.2%)	3,170 (26.4%)	6,402 (53.4%)	
Sometimes/Never	388 (11.4%)	332 (9.7%)	878 (25.7%)	1,817 (53.2%)	
Uninsured	153 (14.2%)	133 (12.3%)	264 (24.5%)	529 (49.0%)	

Medical bills					$\chi^2 = 106.78$ ($p < 0.001$)
No bills	995 (10.1%)	950 (9.6%)	2,497 (25.3%)	5,449 (55.1%)	
Had difficulty	751 (13.5%)	573 (10.3%)	1,608 (28.9%)	2,628 (47.3%)	
Did not have difficulty	2,174 (11.0%)	1,796 (9.1%)	5,254 (26.6%)	10,519 (53.4%)	

Table S2: Descriptive Characteristics of Participants with ASD and ADHD, by Medicaid Expansion and COVID-19 Period

Variable	Expansion Status		COVID-19 Period		Chi2 (p-value)
	Expansion	Non-expansion	Pre-COVID-19	During/Post-COVID-19	
Age group					$\chi^2 = 25.00$ ($p < 0.001$)
Aged 3-5 years	21 (7.0%)	41 (13.7%)	47 (15.7%)	190 (63.6%)	
Aged 6-11 years	168 (10.4%)	172 (10.6%)	389 (24.0%)	889 (54.9%)	
Older than 12 years	249 (10.5%)	225 (26.2%)	623 (26.2%)	1,284 (53.9%)	
Race/Ethnicity					$\chi^2 = 26.09$ ($p = 0.002$)
Hispanic	42 (7.7%)	68 (12.5%)	112 (20.6%)	322 (59.2%)	
White, non-Hispanic	327 (10.9%)	286 (9.6%)	760 (25.4%)	1618 (54.1%)	
Black, non-Hispanic	34 (11.8%)	40 (13.8%)	69 (23.9%)	146 (50.5%)	
Multi-racial, non-Hispanic	35 (7.4%)	44 (9.3%)	118 (24.9%)	277 (58.44%)	
Metro status					$\chi^2 = 11.48$ ($p = 0.01$)
Metropolitan	376 (10.2%)	376 (10.2%)	937 (25.5%)	1,987 (54.1%)	
Non-Metropolitan	62 (10.0%)	62 (10.0%)	122 (19.6%)	376 (60.5%)	

Family structure					$\chi^2 = 21.16$ (p = 0.01)
Two parents, currently married	276 (10.7%)	240 (9.3%)	646 (24.9%)	1,429 (55.2%)	
Two parents, not currently married	26 (9.7%)	23 (8.6%)	81 (30.1%)	139 (51.7%)	
Single parent (mother only)	106 (9.6%)	142 (12.8%)	241 (21.7%)	621 (56.0%)	
Grandparent household	30 (9.2%)	33 (10.1%)	91 (27.7%)	174 (53.1%)	
Education (Highest adult)					$\chi^2 = 4.38$ (p = 0.63)
< HS	12 (10.1%)	16 (13.6%)	35 (29.4%)	56 (47.1%)	
HS or GED	72 (10.4%)	75 (10.8%)	166 (24.0%)	380 (54.8%)	
Some college or more	354 (10.2%)	347 (10.0%)	858 (24.6%)	1,927 (55.3%)	
Insurance type					$\chi^2 = 22.23$ (p = 0.01)
Public only	154 (10.1%)	174 (11.4%)	354 (23.1%)	848 (55.4%)	
Private only	212 (10.5%)	207 (10.2%)	517 (25.5%)	1,092 (53.9%)	
Public and private	56 (9.1%)	38 (6.2%)	157 (25.6%)	363 (59.1%)	
Uninsured	16 (12.7%)	19 (15.1%)	31 (24.6%)	60 (47.6%)	
Federal Poverty Level (FPL)					$\chi^2 = 12.23$ (p = 0.20)
0-199%	173 (10.2%)	194 (11.4%)	400 (23.5%)	936 (55.0%)	
200-299%	78 (11.2%)	71 (10.2%)	158 (22.7%)	390 (54.0%)	
300-399%	61 (10.6%)	56 (9.8%)	158 (27.5%)	299 (52.1%)	
400% or more	126 (9.5%)	117 (8.8%)	343 (25.9%)	738 (55.7%)	
Household language					$\chi^2 = 4.87$ (p = 0.18)
English	424 (10.36%)	415 (10.1%)	1,015 (24.8%)	2,238 (54.7%)	
Other	14 (6.8%)	23 (11.2%)	44 (21.4%)	125 (60.7%)	
Sex of child					$\chi^2 = 4.70$ (p = 0.20)
Male	352 (10.4%)	347 (10.2%)	854 (25.2%)	1,855 (54.2%)	
Female	86 (9.5%)	91 (10.0%)	205 (22.5%)	528 (58.0%)	

Insurance consistency					$\chi^2 = 11.55$ (p = 0.01)
Consistently insured	412 (10.0%)	408 (9.9%)	1,012 (24.6%)	2,276 (54.4%)	
Uninsured/inconsistent	26 (13.7%)	30 (15.8%)	47 (24.7%)	87 (45.8%)	
Insurance benefit coverage					$\chi^2 = 12.23$ (p = 0.20)
Always	201 (10.3%)	209 (10.7%)	469 (24.0%)	1,073 (55.0%)	
Usually	152 (9.3%)	157 (9.6%)	415 (25.4%)	912 (55.8%)	
Sometimes/Never	69 (11.7%)	53 (9.0%)	150 (25.3%)	320 (54.1%)	
Uninsured	16 (13.6%)	19 (16.1%)	25 (21.2%)	58 (49.2%)	
Medical bills					$\chi^2 = 37.79$ ($p < 0.001$)
No bills	125 (9.1%)	150 (11.0%)	301 (22.0%)	794 (58.0%)	
Had difficulty	101 (12.0%)	93 (11.1%)	257 (30.6%)	390 (46.4%)	
Did not have difficulty	212 (10.2%)	195 (9.3%)	501 (24.0%)	1,179 (56.5%)	

Table S3: Full logistic regression results of the model for current medication use, main effects only.

			ASD/ADHD			ASD + ADHD
**Characteristic**	**aOR**	**95% CI**	**p-value**	**aOR**	**95% CI**	**p-value**
Current Medicaid expansion			<0.001			0.9
Non-Expansion	—	—		—	—	
Expansion	0.68	0.60, 0.77		0.98	0.74, 1.30	
COVID-19 period			>0.9			0.4
Pre- COVID -19	—	—		—	—	
During or Post COVID -19	0.99	0.79, 1.25		1.22	0.73, 2.05	
Year	0.94	0.90, 0.99	0.023	0.90	0.81, 1.00	0.055

Age group			<0.001			0.003
Aged 3-5 years old	—	—		—	—	
Aged 6 -11 years old	5.36	3.92, 7.33		2.88	1.45, 5.71	
Older than 12 years old	5.13	3.70, 7.11		3.26	1.65, 6.42	
Sex			0.7			0.4
Male	—	—		—	—	
Female	0.98	0.89, 1.08		0.86	0.60, 1.22	
Race/Ethnicity			<0.001			0.2
Hispanic	—	—		—	—	
White, non-Hispanic	1.63	1.39, 1.90		1.47	0.91, 2.38	
Black, non-Hispanic	1.38	1.14, 1.67		1.39	0.77, 2.52	
Multi-racial, non-Hispanic	1.21	1.00, 1.46		1.02	0.58, 1.78	
Language spoken at home			<0.001			0.6
English	—	—		—	—	
Other than English	0.59	0.43, 0.80		1.21	0.56, 2.59	
Family structure			<0.001			0.4
Two parents, currently married	—	—		—	—	
Two parents, not currently married	0.74	0.60, 0.90		1.10	0.65, 1.86	
Single parent (mother or father)	1.05	0.91, 1.21		1.20	0.82, 1.75	
Grandparent household or other family type	1.34	1.12, 1.61		1.56	0.92, 2.65	
Metro area	1.15	1.00, 1.31	0.047	1.33	0.94, 1.87	0.10

Income relative to federal poverty level			0.017			0.9
0-199% FPL	—	—		—	—	
200-299% FPL	1.12	0.94, 1.33		1.01	0.63, 1.60	
300-399% FPL	1.07	0.90, 1.27		1.21	0.76, 1.90	
400% FPL & more	1.25	1.07, 1.47		1.08	0.70, 1.67	
Parental highest education			0.075			0.6
Less than high school	—	—		—	—	
High school or GED	0.96	0.67, 1.36		1.21	0.60, 2.42	
Some college or technical school	0.81	0.59, 1.12		0.99	0.51, 1.89	
Insurance type			0.053			0.003
Public health insurance only	—	—		—	—	
Private health insurance only	0.81	0.68, 0.96		0.67	0.45, 0.99	
Public and private insurance	0.98	0.83, 1.16		1.34	0.84, 2.16	
Uninsured	0.77	0.30, 1.97		0.80	0.11, 5.75	
Insurance coverage consistency			0.008			0.5
Consistently insured throughout the past year	—	—		—	—	
Currently uninsured or had periods without coverage	0.63	0.45, 0.89		0.72	0.25, 2.05	
Insurance covers needs			0.4			0.4
Always	—	—		—	—	
Usually	0.91	0.81, 1.03		1.28	0.92, 1.77	
Sometimes or never	0.92	0.77, 1.09		0.98	0.59, 1.60	

Uninsured	0.96	0.36, 2.55		0.51	0.05, 5.22	
Able to pay of medical bills			0.11			0.5
No medical or health-related expenses	—	—		—	—	
Had problems paying medical bills	0.95	0.79, 1.15		1.26	0.80, 1.98	
Did not have problems paying medical bills	1.11	0.94, 1.32		1.28	0.85, 1.93	

Abbreviations: CI = Confidence Interval, OR = Odds Ratio

Table S4: Full logistic regression results of the model for current medication use, including the interaction between Medicaid expansion status and the COVID-19 period.

	ASD/ADHD			ASD + ADHD		
**Characteristic**	**aOR**	**95% CI**	**p-value**	**aOR**	**95% CI**	**p-value**
Current Medicaid expansion			<0.001			0.2
Non-Expansion	—	—		—	—	
Expansion	0.57	0.46, 0.70		0.74	0.46, 1.18	
COVID-19 period			0.2			0.8
Pre- COVID -19	—	—		—	—	
During or Post COVID -19	0.84	0.66, 1.08		0.95	0.53, 1.68	
Current Medicaid Expansion x COVID-19 period			0.020			0.11
Expansion x During or Post COVID-19	1.35	1.05, 1.74		1.60	0.90, 2.86	
Year	0.94	0.90, 0.99	0.015	0.90	0.81, 1.00	0.044
Age group			<0.001			0.003

Aged 3-5 years old	—	—		—	—	
Aged 6 -11 years old	5.41	3.99, 7.34		2.96	1.48, 5.91	
Older than 12 years old	5.16	3.75, 7.11		3.33	1.68, 6.61	
Sex			0.6			0.4
Male	—	—		—	—	
Female	0.98	0.89, 1.08		0.87	0.61, 1.23	
Race/Ethnicity			<0.001			0.2
Hispanic	—	—		—	—	
White, non-Hispanic	1.62	1.39, 1.88		1.48	0.91, 2.39	
Black, non-Hispanic	1.37	1.13, 1.66		1.37	0.76, 2.48	
Multi-racial, non-Hispanic	1.20	1.0, 1.45		1.01	0.58, 1.77	
Language spoken at home			<0.001			0.7
English	—	—		—	—	
Other than English	0.59	0.43, 0.80		1.19	0.56, 2.52	
Family structure			<0.001			0.4
Two parents, currently married	—	—		—	—	
Two parents, not currently married	0.74	0.60, 0.90		1.10	0.65, 1.87	
Single parent (mother or father)	1.05	0.91, 1.21		1.19	0.82, 1.73	
Grandparent household or other family type	1.34	1.12, 1.61		1.51	0.90, 2.54	
Metro area	1.15	1.00, 1.31	0.049	1.30	0.93, 1.83	0.13
Income relative to federal poverty level			0.018			0.9

0-199% FPL	—	—		—	—	
200-299% FPL	1.12	0.94, 1.33		1.0	0.62, 1.59	
300-399% FPL	1.07	0.90, 1.27		1.20	0.75, 1.90	
400% FPL & more	1.25	1.07, 1.47		1.09	0.70, 1.69	
Parental highest education			0.077			0.6
Less than high school	—	—		—	—	
High school or GED	0.95	0.67, 1.35		1.18	0.60, 2.32	
Some college or technical school	0.81	0.59, 1.12		0.96	0.51, 1.81	
Insurance type			0.056			0.003
Public health insurance only	—	—		—	—	
Private health insurance only	0.81	0.69, 0.96		0.66	0.44, 0.97	
Public and private insurance	0.98	0.83, 1.16		1.31	0.83, 2.06	
Uninsured	0.78	0.30, 2.02		0.81	0.10, 6.57	
Insurance coverage consistency			0.007			0.6
Consistently insured throughout the past year	—	—		—	—	
Currently uninsured or had periods without coverage	0.63	0.45, 0.88		0.74	0.27, 2.05	
Insurance covers needs			0.4			0.4
Always	—	—		—	—	
Usually	0.91	0.81, 1.02		1.27	0.91, 1.76	
Sometimes or never	0.91	0.77, 1.09		0.97	0.59, 1.60	
Uninsured	0.94	0.35, 2.55		0.49	0.04, 5.61	

Able to pay of medical bills			0.10			0.5
No medical or health-related expenses	—	—		—	—	
Had problems paying medical bills	0.95	0.79, 1.15		1.27	0.81, 2.01	
Did not have problems paying medical bills	1.11	0.94, 1.32		1.28	0.85, 1.92	

Abbreviations: CI = Confidence Interval, OR = Odds Ratio

Table S5: Full logistic regression results for sensitivity analysis, removing the 2020 year data collection.

	Model 1: Main effects			Model 2: Interaction		
**Characteristic**	**aOR**	**95% CI**	**p-value**	**aOR**	**95% CI**	**p-value**
Current Medicaid expansion			<0.001			<0.001
Non-Expansion	—	—		—	—	
Expansion	0.65	0.57, 0.75		0.57	0.46, 0.70	
COVID-19 period			0.7			0.2
Pre- COVID -19	—	—		—	—	
During or Post COVID -19	0.95	0.69, 1.31		0.82	0.59, 1.14	
Current Medicaid Expansion x COVID-19 period						0.053
Expansion x During or Post COVID-19				1.30	1.00, 1.70	
Year	0.95	0.89, 1.02	0.13	0.95	0.89, 1.01	0.12
Age group			<0.001			<0.001
Aged 3-5 years old	—	—		—	—	

Aged 6 -11 years old	4.89	3.54, 6.75		4.93	3.60, 6.75	
Older than 12 years old	4.70	3.34, 6.61		4.72	3.38, 6.61	
Sex			0.8			0.7
Male	—	—		—	—	
Female	0.99	0.89, 1.09		0.98	0.89, 1.08	
Race/Ethnicity			<0.001			<0.001
Hispanic	—	—		—	—	
White, non-Hispanic	1.65	1.40, 1.94		1.64	1.39, 1.93	
Black, non-Hispanic	1.38	1.12, 1.69		1.37	1.11, 1.68	
Multi-racial, non-Hispanic	1.25	1.03, 1.52		1.24	1.02, 1.51	
Language spoken at home			<0.001			<0.001
English	—	—		—	—	
Other than English	0.57	0.42, 0.78		0.57	0.42, 0.78	
Family structure			<0.001			<0.001
Two parents, currently married	—	—		—	—	
Two parents, not currently married	0.75	0.62, 0.92		0.75	0.62, 0.92	
Single parent (mother or father)	1.08	0.93, 1.27		1.09	0.93, 1.27	
Grandparent household or other family type	1.38	1.13, 1.68		1.38	1.13, 1.68	
Metro area	1.16	1.00, 1.34	0.049	1.16	1.00, 1.34	0.052
Income relative to federal poverty level			0.027			0.029
0-199% FPL	—	—		—	—	

200-299% FPL	1.12	0.94, 1.33		1.12	0.94, 1.33	
300-399% FPL	1.11	0.92, 1.32		1.11	0.93, 1.33	
400% FPL & more	1.27	1.08, 1.50		1.27	1.08, 1.50	
Parental highest education			0.2			0.2
Less than high school	—	—		—	—	
High school or GED	0.91	0.63, 1.32		0.91	0.63, 1.31	
Some college or technical school	0.81	0.57, 1.14		0.81	0.57, 1.14	
Insurance type			0.2			0.2
Public health insurance only	—	—		—	—	
Private health insurance only	0.83	0.70, 1.00		0.84	0.70, 1.00	
Public and private insurance	0.99	0.83, 1.19		0.99	0.83, 1.18	
Uninsured	0.84	0.32, 2.17		0.85	0.33, 2.22	
Insurance coverage consistency			0.014			0.013
Consistently insured throughout the past year	—	—		—	—	
Currently uninsured or had periods without coverage	0.64	0.45, 0.91		0.64	0.45, 0.91	
Insurance covers needs			0.6			0.6
Always	—	—		—	—	
Usually	0.92	0.82, 1.04		0.92	0.82, 1.04	
Sometimes or never	0.99	0.82, 1.19		0.99	0.82, 1.19	
Uninsured	0.94	0.35, 2.57		0.92	0.33, 2.57	
Able to pay of medical bills			0.053			0.049

No medical or health-related expenses	—	—	—	—
Had problems paying medical bills	0.90	0.74, 1.10	0.90	0.74, 1.10
Did not have problems paying medical bills	1.10	0.91, 1.33	1.10	0.91, 1.33

Abbreviations: CI = Confidence Interval, OR = Odds Ratio

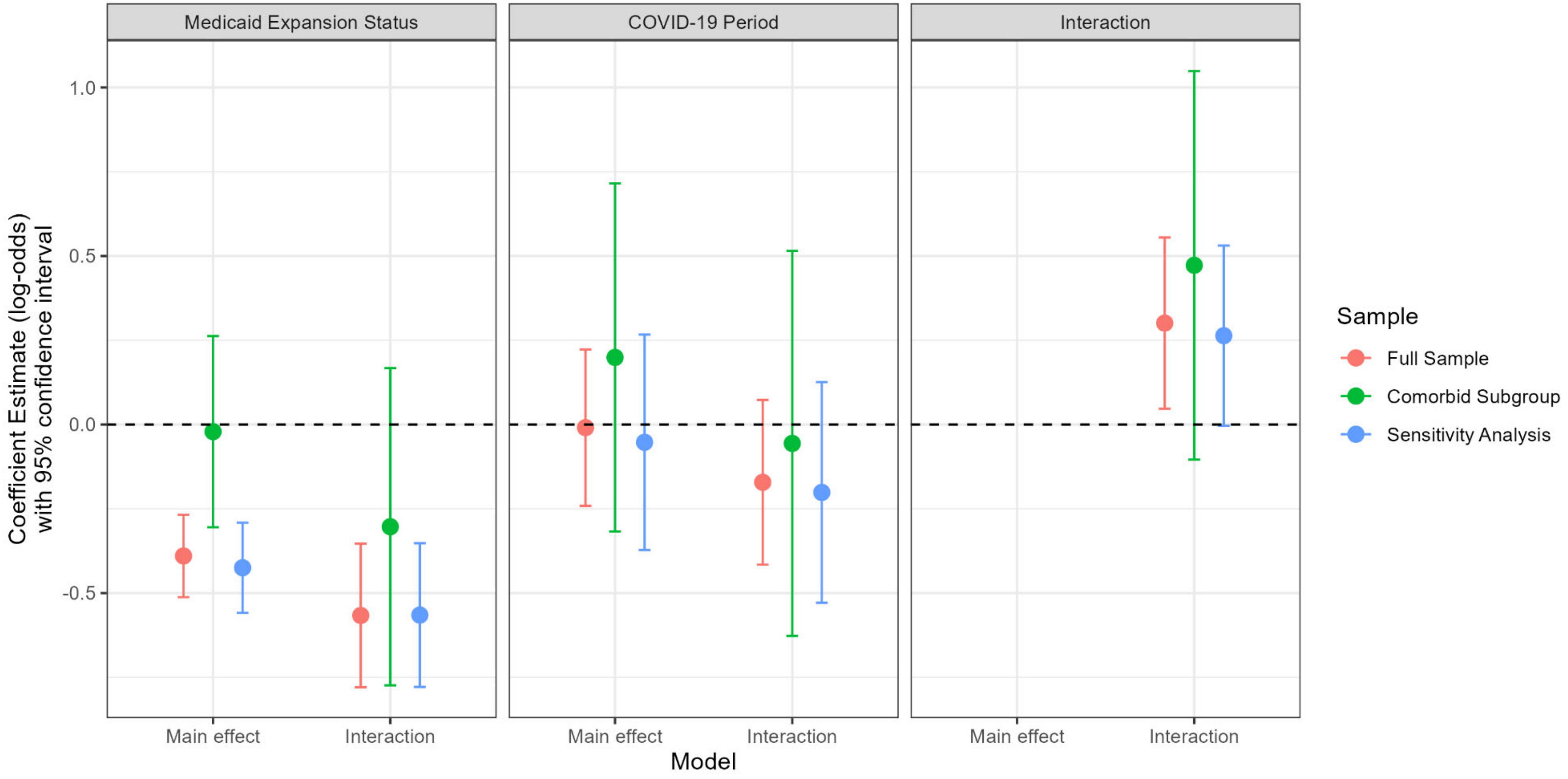

Figure S1: Logistic regression coefficients for Medicaid expansion, COVID-19 period, and their interaction in the full sample, comorbid subgroup, and sensitivity analysis.
